# Evaluation of the Copan ESwab Transport System for Viability of Pathogenic Fungi by Use of a Modification of Clinical and Laboratory Standards Institute Document M40-A2

**DOI:** 10.1128/JCM.01481-17

**Published:** 2018-01-24

**Authors:** Bharat Gandhi, Richard Summerbell, Tony Mazzulli

**Affiliations:** a Department of Microbiology, Mt. Sinai Hospital and University Health Network, Toronto, Ontario, Canada; b Sporometrics, Toronto, Ontario, Canada; c Dalla Lana School of Public Health, University of Toronto, Toronto, Ontario, Canada; d Department of Laboratory Medicine and Pathobiology, University of Toronto, Toronto, Ontario, Canada

**Keywords:** CLSI M40-A2, Copan ESwab system, pathogenic fungi, photometric inoculum adjustment

## Abstract

The Copan ESwab system was evaluated for its ability to maintain the viability of pathogenic fungi. Tests followed the Clinical and Laboratory Standards Institute (CLSI) document M40-A2 roll-plate method at room and refrigerator temperatures. A system was devised for standardizing homogeneous inoculum suspensions of variously sized conidia and sporangiospores of filamentous fungi. A total of 19 clinical and reference strains were standardized to a 0.5 McFarland turbidity standard with a simple photometer. Corresponding optical densities were measured with a spectrophotometer. Colony counts equal to or greater than those seen at time zero were obtained for the entire test panel. Results indicate that the Copan ESwab system effectively maintains prevalent opportunistic fungal organisms for at least 48 h.

## INTRODUCTION

The Clinical and Laboratory Standards Institute (CLSI) document M40-A2 describes methods for assessing the ability of transport medium devices to maintain potentially pathogenic microorganisms in a viable condition for up to 48 h during transport at room temperature (RT) (20°C to 25°C) and at refrigerator temperature (FT) (2°C to 8°C) (1). Typically, the standard panel of bacterial strains chosen to validate the transport media represents a diverse group of bacterial challenge organisms. However, the panel does not include any fungal pathogens.

No formally published data are available on the viability of filamentous fungi during transport using swab-based systems. Preliminary data, however, were presented in 2010 in a poster at the American Society for Microbiology 110th General Meeting (2). The 2010 study was conducted only at room temperature.

In this study, 100 μl of standardized inoculum was delivered to each flocked swab directly, and 10-μl aliquots were dispensed onto plates of medium for control colony enumeration. Contrary to the recommendations of Snyder et al. (1), who also tested the Copan ESwab kit, CLSI recommends allowing the flocked swabs to absorb material from the well of a microtiter plate containing 100 μl of inoculum. It is recommended that swabs be held at RT and FT. The current investigation was designed to adopt CLSI-recommended methods to allow for a canonical evaluation of the Copan ESwab transport system (Copan Diagnostics Inc., Murrieta, CA) for fungal pathogens described below. Because of labor, resource intensity, and incubator space restrictions, these types of studies are seldom conducted.

(The results of this work were partially presented at the 117th General Meeting of the American Society for Microbiology, New Orleans, LA, 3 to 6 June 2017.)

## MATERIALS AND METHODS

A test panel was prepared that consisted of frequently isolated organisms in the clinical laboratory and a comprehensive selection of clinically important opportunistic pathogens. Viability studies followed CLSI M40-A2 methods, with minimal modifications that were necessary because of the physical properties of fungal inocula. Most important, as described below, methods were developed to make uniform suspensions of hydrophobic fungi such as Aspergillus spp. (3); in addition, the problem of accurately counting rapidly expanding colonies in growth studies was addressed. Techniques for suspension standardization were modified and validated to ensure reliable test evaluation. Specifically, a fast and practical photometric procedure for cell density standardization was developed. This study is the first to use these methods.

### Study isolates.

A test panel of yeasts and filamentous fungi was assembled, including quality-control reference strains, clinical isolates cultured from swab transport systems, and superficial specimens in routine high-volume clinical microbiology laboratories. This collection of 19 test isolates consisted of 5 yeast reference isolates (Candida albicans ATCC 10231, Candida krusei ATCC 6258, Candida guilliermondii ATCC 6260, Candida glabrata ATCC 66032, and Cryptococcus neoformans ATCC 66031), 3 reference dermatophytes (Trichophyton mentagrophytes ATCC 9533, Trichophyton tonsurans ATCC 28942, and Trichophyton rubrum ATCC 28188), 5 opportunistic hyaline molds (Aspergillus niger, Aspergillus fumigatus, Lecythophora sp., Fusarium solani, and Trichosporon sp.), 3 Zygomycetes (Lichtheimia corymbifera, Mucor circinelloides, and Rhizopus microsporus), and 3 dematiaceous molds (Curvularia clavata, Phialophora americana, and Alternaria alternata). Identification of the opportunistic molds at the species level was confirmed by an independent reference laboratory.

### Inoculum preparation.

Strains previously stored at −80°C in cryopreservative fluid were subcultured twice before testing. First, they were cultured on Sabouraud dextrose agar (Biomedia Unlimited Ltd.) to ensure viability and purity, and then they underwent a second subculture on potato dextrose agar (Biomedia Unlimited Ltd.) to optimize sporulation. All plates were incubated aerobically at 30°C until confluent growth was observed, with incubation time ranging from ∼2 to 14 days. The inoculum was prepared by zeroing 5.0 ml (0.85%) of sterile saline (Oxoid Inc.) containing 1 drop of Tween 20 (Biomedia Unlimited Ltd.), as per CLSI M38-A2 (3), in a spectrophotometer (WP-100DPlus, Walter Products Inc.) at a mark scribed on the tube. The addition of Tween surfactant is not required to produce uniform suspensions of bacteria (1), but it is used to prepare uniform suspensions of hydrophobic fungi (3). This slightly modified sterile saline diluent was poured onto a mature culture plate. A mature culture is when point inoculation in the center of a plate produces only one colony that spreads outward. Once colored powdery conidia are observed visually after ∼3 days, the mold is sufficiently mature to carry out laboratory investigations. Initially, organisms were identified to the genus level by way of Scotch tape and tease-mount preparations. After initial evaluation of these techniques to confirm their reliability and reproducibility, we felt that it was not necessary to continue to perform these tests. Propagules were harvested by gently rubbing the surface of the plate with a swab. The coarse suspension was pipetted back into the original empty tube, vortexed, and allowed to sit for 30 min. The top portion of the suspension was separated from the heavier hyphal sediment by means of a sterile transfer pipette. This supernatant was then manually adjusted to a 0.5 McFarland turbidity standard using a Wickerham card to produce ∼1 × 10^6^ to 5 × 10^6^ CFU/ml (4). Fine adjustments were performed on the DensiChek Plus photometric device to 0.5 McFarland (between 0.45 and 0.55 McFarland). The corresponding absorbance and percent transmission values were obtained using round glass tubes containing saline because they came with screw caps to contain any aerosols and fitted well into the spectrophotometer. As per the manufacturer's directions, a line was scribed near the top of the tube so that the absorbance/transmission readings could be determined at the same spot as the zeroing determined at 530 nm using the spectrophotometer. The readings were recorded for comparison with published values (4).

### CLSI M40-A2 roll-plate method.

Inocula were prepared for the modified roll-plate method. For each prepared inoculum tube adjusted to the 0.5 McFarland turbidity standard (∼1 × 10^6^ to 5 × 10^6^ CFU/ml), three 10-fold dilutions were performed at concentrations of 10^5^, 10^4^, and 10^3^ CFU/ml. Using an Eppendorf repeater pipette, 100-μl aliquots of the dilutions were dispensed into a round-bottomed 96-well microtiter plate in triplicate for each organism suspension and dilution. Swabs were immersed into the dispensed suspension, absorbed for 10 s, and transferred into the ESwab device containing 1 ml of liquid Amies transport medium. One set of inoculated swabs was held at RT (20°C to 25°C) and a duplicate set at FT (2°C to 8°C). After 0, 24, and 48 h, the swabs were removed. Initially, three swabs were tested at time zero for each organism and each dilution by removing the selected swabs from the transport device after ∼5 to 15 min. The ESwab transport devices, including the swab, were vortexed to express residual fluid from the swabs and discarded. The transport devices were vortexed again for 5 s. For 10^5^ concentrations, to minimize potentially confluent growth of rapidly extending fungal colonies, only 50-μl aliquots (instead of 100-μl aliquots) were pipetted and then evenly distributed with a sterile 10-μl loop on three plates for each duplicate set.

The resulting colony counts from each set of three plates were multiplied by 2 to give equivalent counts for 100 μl inoculum. For inocula at a concentration of 10^4^, 100 μl was pipetted and evenly distributed on two plates for each duplicate set; colony counts were then totaled. For inocula at10^3^ concentration, 100 μl was pipetted on a single plate; since two parallel inocula were tested, this test was effectively done in duplicate. The plates were then sealed with Parafilm, incubated at 30°C aerobically, and observed for growth each day until distinct colonies formed on the entire plate. Once growth was observed, the colonies were counted visually by marking on the reverse side of the plate with a black felt-tip pen and using a mechanical counter. After growth is seen, fungal colonies can be counted (countable colonies) similar to bacterial colonies.

Zygomycetes, however, grow rapidly and confluently within 2 days. They also do not produce a darkening at the point where the colony originates on the reverse side of the plate. This makes it difficult to distinguish individual colonies and, therefore, to count them. CFU were averaged and tabulated. Similar steps were followed for growth controls by transferring the working inoculum onto plates.

## RESULTS

The isolates were tested once using one ESwab lot number. Even at the highest dilution, it was possible to produce colony counts at 24 and 48 h that were similar to those at 0 h (Table 1). All isolates yielding 0-h counts of ≥5 CFU at a given inoculum dilution also yielded 24- and 48-h counts of ≥5 CFU at RT and FT. Most of the test isolates produced CFU in the 30 to 300 range at the 10^4^ dilutions. As detailed above, this enumeration was facilitated in the more rapidly growing fungi by using several plates and reducing the volume of inoculum. Two of the three Zygomycetes, i.e., Mucor circinelloides and Lichtheimia corymbifera, and one dematiaceous mold, i.e., Curvularia clavata, produced countable colonies from only the 10^5^ dilutions; these were species with notably broad-spreading colonies. Rhizopus microsporus produced colonial growth that was not countable even under those conditions, although it remained vigorously viable. All 19 organisms tested from 0.5 McFarland-adjusted suspensions produced transmission values of ∼71% to 80% on the spectrophotometer using the saline-Tween 20 diluents, similar to values cited in the literature of ∼68% to 82% transmission (4).

**TABLE 1:** Recovery of pathogenic fungi after 24 and 48 h

Organism	Recovery at concentration of^*a*^:														
	10^5^ CFU/ml at (h):					10^4^ CFU/ml at (h):					10^3^ CFU/ml at (h):				
	0	24		48		0	24		48		0	24		48	
	RT	4°C	RT	4°C	RT	RT	4°C	RT	4°C	RT	RT	4°C	RT	4°C	RT
Yeast															
Candida albicans	723	842	822	>500	>500	171	133	283	152	>500	23	20	31	17	>500
Candida krusei	>500	>500	>500	>500	>500	297	208	>500	265	>500	34	34	295	22	>500
Candida guilliermondii	>500	>500	>500	>500	>500	456	387	>500	307	>500	98	67	294	75	>500
Candida glabrata	>500	>500	>500	466	>500	320	152	314	236	>500	38	25	79	31	321
Cryptococcus neoformans	>500	>500	>500	450	>500	207	241	309	306	>500	33	44	72	37	315
Dermatophyte															
Trichophyton mentagrophytes	332	375	378	405	401	61	28	26	20	33	8	7	6	9	11
Trichophyton tonsurans	441	>500	>500	399	375	39	37	32	41	34	4	3	4	2	2
Trichophyton rubrum	>500	355	333	346	431	229	43	35	20	49	14	8	5	4	19
Hyaline/saprobe															
Aspergillus niger	388	>500	>500	>500	>500	80	81	112	92	80	13	11	12	13	12
Lecythophora sp.	400	>500	>500	360	480	100	100	46	62	81	8	9	5	10	9
Fusarium solani	>500	>500	>500	>500	>500	269	260	249	269	274	32	25	34	29	32
Trichosporon sp.	134	232	100	138	143	31	27	28	31	33	11	5	4	5	6
Aspergillus fumigatus	>500	>500	>500	>500	>500	260	197	239	264	213	15	13	19	14	13
Zygomycete															
Lichtheimia corymbifera	62	61	78	63	75	12	7	7	12	7	1	2	1	1	0
Mucor circinelloides	112	120	100	46	41	14	25	15	20	6	3	2	3	3	0
Rhizopus microsporus	160	NC	NC	NC	NC	25	NC	NC	36	NC	2	3	2	2	2
Dematiaceous mold															
Curvularia clavata	74	67	80	81	45	18	14	15	13	10	2	1	2	1	1
Phialophora americana	>500	>500	450	470	321	106	98	77	102	54	16	14	6	7	6
Alternaria alternata	>500	>500	>500	>500	>500	230	305	300	187	199	16	24	28	25	18

a Average of triplicate tests in CFU/100 μl. NC, not countable; RT, room (ambient) temperature; 4°C, refrigerator temperature.

## DISCUSSION

Typically, transport media systems are used for collection of fungi from primary specimens, which include ear, throat, vagina, urethra, penis, and wounds other than dermatophytic specimens, such as skin, nails, and hair. However, a scalp specimen is frequently received on swab transport media for KOH examination and culture in the laboratory. There are many studies of this method versus the traditional method of scrapings received in black paper yielding comparable results.

From a scientific standpoint, it was important to validate a broad spectrum of pathogenic fungi to assess the viability by this mode of transport to allow for a successful work up. Fungi are eukaryotic organisms with cells that are mostly relatively large and robust compared with bacterial cells. Nonetheless, it cannot be taken for granted that these organisms, when transported from bedside to laboratory via standard specimen collection methods, will retain viability. In addition, whether their relatively large sizes or their physical properties, such as hydrophobic exteriors or mucoid coatings, might interfere with detachment from swab-based transport systems is unknown. In the current study, potentially clinically important fungi with a wide range of physical propagule characteristics (including hydrophobic and hydrophilic cell walls, unicellular and multicellular propagules, smooth and echinulate integuments), cell colors (hyaline, melanized), and colony growth rates and forms (discretely colonial, diffuse) were tested. Inoculum standardization and colony-counting methods were modified to accommodate these differences. More important, these accommodations were designed to produce an equivalent to the bacterial CLSI standard for clinically important fungi. The criteria set by the newly revised second edition of the CLSI M40-A2 standard for the roll-plate method states that for compliance of viability, any specimen held at 4°C and RT should yield ≥5 CFU after a specified holding period. Results suggest that the Copan ESwab transport system is able to maintain and recover a broad range of fungi after 48 h at 4°C and RT. However, the one limitation of this study was that it was not performed on actual clinical specimens that might be harboring yeast or filamentous fungi pathogens. Therefore, the organism counts in a clinical specimen might be different from those contrived in this study. Recovery at 24 and 48 h might vary depending on organism load in a specimen. This supports the CLSI document M40-A2 standard for validation using the modified procedure. The CLSI-recommended spectrophotometric methods of standardization for conidial and sporangiospore suspensions are difficult to perform with fungi because variabilities in spore color and size cause different fungi to have different optical densities at the same levels of CFU (4). Species-dependent standardization processes are not routinely used in microbiology laboratories and are not familiar to lab personnel. Several studies have compared widely used methods such as cell counting by hemocytometer, which is a procedure stipulated in the EUCAST E.DEF9.1 standard (5–7), but few studies have tested photometers as alternatives for hemocytometric and spectrophotometric measurements (7, 8).

In our study, density standardization was found to be streamlined by using a simple photometric device, DensiCheck Plus, for inoculum adjustments; it proved to be a very good alternative to more expensive and less user-friendly spectrophotometers. It may be imperative to address these issues in a further study; however, we are confident that the ESwab is suitable for fungal viability and transport in accordance with the CLSI standard for clinically important fungi.
